# Veterinary parasitologists: the time has come to talk about the use of the expressions “Protozoan” and “Protista”

**DOI:** 10.1590/S1984-29612025021

**Published:** 2025-04-14

**Authors:** José Reck, Alynne da Silva Barbosa, Huarrison Azevedo Santos, Filipe Dantas-Torres, Marcos Rogério André, George Rego Albuquerque

**Affiliations:** 1 Colégio Brasileiro de Parasitologia Veterinária – CBPV, Jaboticabal, SP, Brasil; 2 Instituto de Pesquisas Veterinárias Desidério Finamor – IPVDF, Secretaria de Agricultura do Estado do Rio Grande do Sul – SEAPI, Eldorado do Sul, RS, Brasil; 3 Universidade Federal Fluminense – UFF, Niterói, RJ, Brasil; 4 Universidade Federal Rural do Rio de Janeiro – UFRRJ, Seropédica, RJ, Brasil; 5 Instituto Aggeu Magalhães, Fundação Oswaldo Cruz – FIOCRUZ, Recife, Brasil; 6 Universidade Estadual Paulista "Júlio de Mesquita Filho" – UNESP, Jaboticabal, SP, Brasil; 7 Universidade Estadual de Santa Cruz – UESC, Ilhéus, BA, Brasil

**Keywords:** Eukaryote, tree of life, phylogeny, parasite, systematic, Eucariotos, árvore da vida, filogenia, parasito, sistemática

## Abstract

The classification of eukaryotic organisms has evolved significantly over the past years. For a long time, the five-kingdom model proposed in 1969, which included the kingdoms Monera, Protista, Fungi, Plantae, and Animalia, dominated biological classification. However, recent advances in molecular biology, particularly phylogenomic studies, have challenged this classification as it does not accurately represent the evolutionary patterns of a vast diversity of organisms, especially those formerly known as protozoa. Currently, Protista is no longer considered a valid taxon, as the organisms previously classified in this group are highly divergent and not monophyletic. Modern approaches now classify eukaryotes into several supergroups, with "protozoa" now dispersed among different groups. For example, parasites once grouped as "protozoa," such as *Babesia* (Apicomplexa), *Trypanosoma* (Euglenozoa), and *Entamoeba* (Evosea), are now placed into distant branches of the tree of life and within different supergroups. Although this supergroup classification may change in the coming years, it provides a more accurate representation of evolutionary relationships among eukaryotes. However, this issue has not been adequately discussed by the veterinary parasitology community. This article advocates revisiting these terms in light of modern classification systems to ensure a more accurate and biologically realistic terminology that reflects current knowledge.

## Resumo

A classificação dos organismos eucarióticos evoluiu significativamente nos últimos anos. Por muito tempo, o modelo de cinco reinos proposto em 1969, que incluía os reinos Monera, Protista, Fungi, Plantae e Animalia, dominou a classificação biológica. No entanto, avanços na biologia molecular, desafiaram essa classificação, pois ela não representa o padrão evolutivo dos organismos, especialmente aqueles anteriormente conhecidos como protozoários. Atualmente, o termo Protista não é considerado um táxon válido, pois os organismos anteriormente classificados neste grupo são altamente distintos e não são monofiléticos. Abordagens modernas classificam os eucariotos em vários supergrupos, nos quais os "protozoários" são colocados em diferentes grupos. Por exemplo, parasitos anteriormente classificados como "protozoários", como *Babesia* (Apicomplexa), *Trypanosoma* (Euglenozoa), *Entamoeba* (Evosea), são agora alocados em ramos distantes da árvore da vida e em diferentes supergrupos. Essa classificação de supergrupos oferece uma representação mais precisa das relações evolutivas entre os eucariotos e evidencia que a evolução (especialmente os organismos unicelulares) segue um padrão complexo de diversificação. Contudo, este tópico ainda não foi adequadamente discutido pela comunidade de parasitologistas veterinários. Este artigo propõe a revisão desses termos tendo em vista sistemas de classificação modernos, para garantir que parasitologistas veterinários e estudantes adotem uma terminologia mais precisa e biologicamente realista.

The pioneering microscopist and founding father of microbiology Anton Van Leeuwenhoek was the first to observe organisms that were invisible to the naked eye (referred to as animalcules), back in the 17th century. He is credited to be the first to observe the organisms which were later thought to be either “protists” (single-celled eukaryotes) or prokaryotes (archaea and bacteria) (Fred, 1933; Corliss, 2002).

In the middle 18th century, Carl Linnaeus (1707-1778), who created the Linnaean system, which is the core of taxonomic nomenclature we still use today (Whittaker, 1969), described several species of ciliates and other unicellular heterotrophic microeukaryotes later identified as “protozoan” (Linnaeus, 1767). His contemporary Otto Friedrich Müller introduced species of “protozoan” to the binomial nomenclature system in his book *Animalcula infusoria et marina* (1786). In 1818, Georg August Goldfuss introduced “Protozoa”, meaning “primordial animals”, as a class of the kingdom Animalia.

As we all have learned in school, living organisms have been grouped in kingdoms for centuries. In fact, the kingdom concept has been present since the first studies of Carl Linnaeus (1707-1778), which have classified living organisms into two kingdoms: *Regnum Animale* (Animal kingdom) and *Regnum Vegetabile* (Plants kingdom). Between the 18^th^ and the late 19^th^ century, the Linnaeus’ two kingdom model was widely adopted.

In 1860, John Hogg proposed a third kingdom, “Protoctista”, to group “primitive” forms of life. Studies by the naturalist Ernst Haeckel between 1866 and 1894 pointed out the demand of a kingdom specific for unicellular organisms. Haeckel proposed the name “Protista” for this third kingdom (Corliss, 1975; Fenchel, 1987; Schmidt & Roberts, 2009).

In the 1930s, the biologist Herbert Copeland argued the need to expand the organism classification to four kingdoms (Copeland, 1938). In 1950s, Copeland’s seminal work consolidated the four kingdom model as a means to have kingdoms specific for bacteria and unicellular eukaryotes (Copeland, 1956). At that time, this model was universally accepted; however a few years later, Robert Whittaker began discussing the need to improve it, especially in order to better classify the unicellular eukaryotes (Whittaker, 1959). In the late 1960s, Whittaker's studies formally led to the proposition of the classical five-kingdom model, consisting of Monera, Protista, Fungi, Plantae, and Animalia (Whittaker, 1969).

Currently, scientists employ several tools available to describe the organisms and classify them into related groups. However, from one perspective, morphology (*via* optical and electron microscopy), and/or genetics (such as rDNA, multigene, and genomics analyses) are the methods mostly used for species description. On the other hand, a significant number of scientists have adopted phylogenetics/phylogenomics to reconstruct natural relationships among organisms and classify them into groups (Dayrat, 2005).

At this point, it may be also useful to clearly define some terms widely used for the study of living organisms. Systematics is the scientific study of biological diversity and evolutionary relationships among organisms. Taxonomy focuses on discovering, describing, and naming species, providing the foundation for understanding biodiversity by defining and delimiting species. Nomenclature refers to the standardized system of assigning scientific names to organisms according to established rules, ensuring consistency and stability in species naming. Classification is the process of organizing species into a hierarchical framework based on shared characteristics, grouping them into *taxa* such as genera, families, and orders. Phylogenetics is the study of evolutionary relationships among organisms, typically using molecular data to construct phylogenetic trees. Phylogenomics extends this approach by incorporating large-scale genomic data, allowing for a more detailed resolution of the evolutionary web. The emerging field of integrative taxonomy combines multiple methods - including morphology, genetics, ecology, development, and behavior - to achieve a more comprehensive understanding of species diversity.

The vast amount of data generated by molecular methods in recent years has dramatically altered some traditional concepts, creating a need for novel and more complex models to explain evolutionary history of organisms. In this context, the five kingdom model appears to be insufficient to accurately represent the evolution of the great biodiversity of living organisms. Firstly, the marked differences between Eubacteria and Archaebacteria made it unacceptable to group them into the same group, Monera (Woese et al., 1990).

Moreover, in the last 30 years, several phylogenetics/phylogenomics studies showed that several organisms formerly classified in the kingdom Protista are highly dissimilar, and they are, indeed, paraphyletic. This raised various debates on the validity of “Protista” as a taxon, as well as about the use of the term “protozoa” as a formal designation of a group of organisms. Also, it raises issues about the classical ways to classify living organisms (Cavalier-Smith, 2003; Adl et al., 2012). Indeed, in a consensus paper of several authors of the International Society of Protistologists (an association of scientists devoted to research on single-celled eukaryotes), Adl et al. (2005) clearly stated that “Protista” and “Protoctista” are no longer formally recognized as taxa. While it is now widely accepted that “Protista” and “Protoctista” are no longer formal taxonomic groups, the evolving classification of “protozoa” is not a consensus, and it seems rarely taught in veterinary parasitology. In fact, the term “protozoa” is still widely used for unicellular eukaryotes that are not animals, plants, or fungi. The aim of this article is to draw the attention of veterinary parasitology researchers, lecturers and professors about the advancements in the higher classification of living organisms, with emphasis on those previously classified in the kingdom Protozoa or Protista.

Instead of being classified into kingdoms, eukaryotes are now grouped into super-groups (Adl et al., 2005, 2012, 2019; Burki et al., 2007, 2020), which largely correspond to clades inferred from phylogenomic studies. Recent descriptions of the tree of life classified eukaryotes into a variety of super-groups (mostly monophyletic) and some additional ungrouped taxa (e.g., Ancoracysta, Picozoa, Malawimonadida, and Ancyromonadida) (Burki et al., 2020). As expected, this modern super-group classification does not correspond to the placement of all “protozoa” in a single group.

Nonetheless, it is important to state there are a lot of open questions regarding the evolution and phylogeny of eukaryotes and, therefore, in their classification (Adl et al., 2005, 2012, 2019; Burki et al., 2007, 2020). Thus, further changes into the higher classification of eukaryotes are expected in the coming years. But why do these classification models and taxonomic nomenclature evolve? Scientists are constantly working to classify organisms, whether for didactic purposes, to understand organism relationships, or to unravel the tree of life. Thus, these various models and terms used to classify organisms are the result of cumulative research data, including fossils, ultrastructural, and genomic information. Moreover, phylogenomics is generating a vast amount of data, which periodically reshapes our understanding of the evolution of organisms and their relationships.

**Figure 1:**
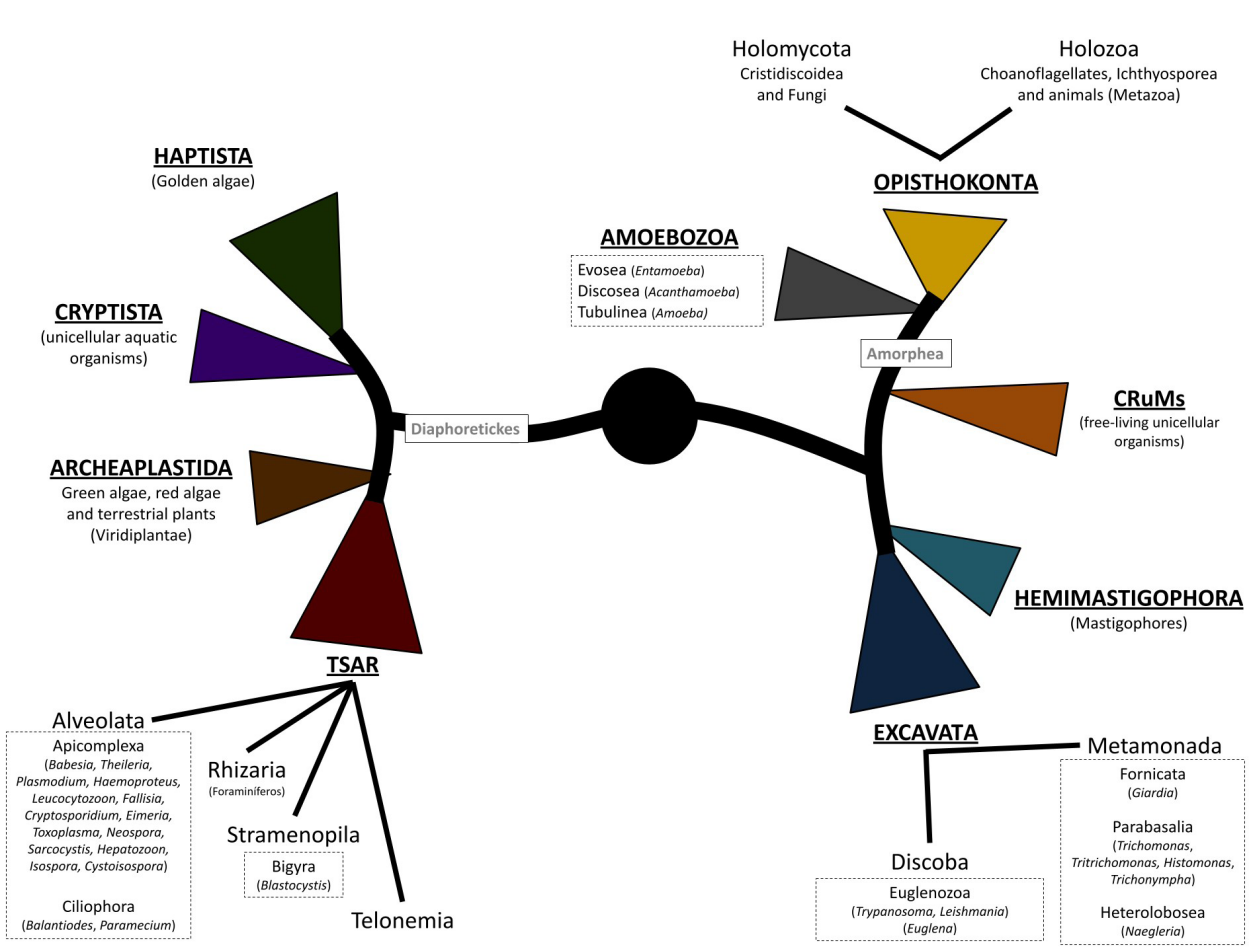
Drawing of a tree showing some of the major currently accepted super-groups of living organisms. This schematic representation was freely drawn by the authors, mostly based on the scheme and topology of the tree of life from Burki et al. (2020). Here, some controversial/orphan groups, not classically associated with parasitism of animals, were intentionally omitted. The schematic tree highlights super-groups in which most living eukaryotes are included. To facilitate the understanding of our major audience (veterinary parasitologists and veterinary students), we included in dashed boxes the current taxonomic position of the most important genera (and the Phylum they belong) of parasites of animals formerly classified as “protozoa” or “Protist”. Amorphea and Diaphoretickes are no-rank classifications proposed by Adl et al. (2019) which denote the evolutionary relationship among the super-groups. All distances are merely illustrative and do not indicate any genetic relationship.

In order to facilitate an understanding of these novel models, in Figure 1, we drew a tree of life showing some of the major currently accepted supergroups of living organisms, mostly based on the scheme and topology proposed by Burki et al. (2020). We arbitrarily omitted some controversial/orphan groups, not classically associated with parasitism of animals. The schematic tree highlights supergroups in which most of living eukaryotes are included: Ophistokonta, Amoebozoa, CRuMs, Hemimastigophora, Excavata, Archeaplastida, Cryptista, Haptista, and TSAR (an acronym derived from the first letters of its four constituent clades: Telonemia, Stramenopiles, Alveolata and Rhizaria). The current taxonomic position of the most important genera (and the phyla they belong to) of parasites formerly classified in the kingdom Protista were shown in dashed boxes. As one can see, parasites of the phylum Apicomplexa (such as those from the genera *Babesia, Theileria, Cytauxzoon, Plasmodium, Haemoproteus, Leucocytozoon, Fallisia, Cryptosporidium, Eimeria, Toxoplasma, Neospora, Sarcocystis, Hepatozoon, Isospora, Cystoisospora*) as well as those belonging to the phylum Cilliophora (genus *Balantioides*) are grouped into the clade Alveolata, which is part of the TSAR super-group. Other well-known animal parasites, such as those from the genera *Trypanosoma* and *Leishmania* of the phylum Euglenozoa, are grouped into the clade Discoba, belonging to the Excavata supergroup. Other branch of Excavata supergroup, the clade Metamonada, includes some well-studied parasites, such as those of the phyla Fornicata (genus *Giardia*), Parabasalia (genera *Trichomonas, Tritrichomonas, Histomonas*), and Heterolobosea (genus *Naegleria*). Some of the organisms generally known as amoebas, such as those from genera *Entamoeba*, *Acanthamoeba* and *Amoeba* cluster into Amoebozoa supergroup. The Metazoa (animals) are grouped into the Opisthokonta supergroup, together with the choanoflagellates (a group of free-living unicellular and colonial flagellate eukaryotes formerly classified as “Protista”). The schematic tree of living organisms provided here is merely illustrative to bring this subject into the spotlight for discussion among veterinary parasitologists. Thus, detailed classification of each of these supergroups is beyond our scope. For a comprehensive review of the eukaryotic supergroups, please see Burki et al. (2020). Controversially, it is important to mention here that certain authors suggest that “Protozoa“ (as well as Chromista) may still be utilized as a “paraphyletic kingdom” (i.e. rejecting the use of monophyly as a guiding principle) (Ruggiero et al., 2015). This “atypical paraphyletic kingdom” would encompass several infra-kingdoms and superphyla, which may correspond to some of the supergroups and phyla depicted in Figure 1.

More than 50 years have elapsed since the proposition of the currently outdated five-kingdom model of Whittaker, but this model is still widely used in most veterinary parasitology courses worldwide. At this time, perhaps we should quote Simpson & Roger (2004), who pointed out that “Protist is a grab-bag for all eukaryotes that are not animals, plants or fungi”. So, why are the five-kingdom model and the kingdom Protista still in use in veterinary parasitology courses and literature? Maybe veterinary parasitologists are still nostalgic and reluctant to abandon use of Protista, Protoctista or Protozoa as formal taxonomic groups. Or perhaps, at least a part of them were unaware about the current taxonomic classification. Nonetheless, it is time for the veterinary parasitology community to review the use of this outdated classification in our textbooks. Instead of writing “*Babesia bovis* is a protozoan”, we should use “*Babesia bovis* is an unicellular eukaryotic parasite of the Phylum Apicomplexa”. Perhaps the classification “Species *Leishmania infantum*, Family Trypanosomatidae, Order Trypanosomatida, Class Kinetoplastea, Phylum Euglenozoa, Kingdom Protista”, should be updated to “Species *Leishmania infantum*, Family Trypanosomatidae, Order Trypanosomatida, Class Kinetoplastea, Phylum Euglenozoa, Supergroup Excavata”. So why do we still teach parasitology within the five kingdoms model? For those concerned how to explain for parasitology students the complexity of supergroups and novel models of the tree of living organisms, we cannot remain attached to old concepts because of their pedagogical and rhetorical utility rather than the biological realism they may reflect (Simpson & Roger, 2004). For an interesting discussion on teaching methods and the evolution of eukaryotes, please see Pereira & Boll (2021).

The use of terms “protists” (= single-celled eukaryotes, which includes algae) and “protozoan” (= single-celled eukaryotes, predominantly non-filamentous heterotrophic species) are still in use in the field of “protistology”, but only as informal terms and do not represent valid taxa in the current classification of eukaryotes (Adl et al., 2019). As our understanding of evolution of eukaryotic organisms has advanced significantly since these terms were first coined, it is prudent, at every time, to consider if their use in veterinary parasitology denotes an informal historic expression or the valid taxonomy. While adopting the modern eukaryotic classification in veterinary parasitology textbooks and classes may require time and effort, this transition will effectively ensure an up-to-date education for veterinary students, researchers, and practitioners, in line with most recent scientific advancements in our discipline. The use of up-to-date classification and terminology in veterinary parasitology ensures scientific integrity and aligns with the dynamic nature of biology and taxonomy. It also recognizes that scientific knowledge is ever evolving and prioritises biological realism over historical convenience.

We concluded that it is time to engage in discussions and teachings about the current understanding of eukaryote evolution, the position of “protozoa” in the tree of life, and the acceptance of the invalidity of 'Protista' as a formal taxon. The discussion presented here should be brought to scientific meetings and classrooms. Finally, the questions raised here should be shared with all our colleagues to foster a broader debate.
